# The Relationship between Lifestyle, Mental Health, and Loneliness in the Elderly during the COVID-19 Pandemic

**DOI:** 10.3390/healthcare12090876

**Published:** 2024-04-23

**Authors:** Daiana Meregalli Schütz, Tainá Rossi, Nathalia Saraiva de Albuquerque, Dalton Breno Costa, Jéssica Santos Machado, Larissa Fritsch, Natacha Gosmann, Raul Costa Mastrascusa, Natália Sessegolo, Vitória Rodrigues Bottega, Luis Eduardo Wearick-Silva, Carmen Moret-Tatay, Francesco Della Gatta, Tatiana Quarti Irigaray

**Affiliations:** 1 Pós-Graduate Program in Psychology, Pontifícia Universidade Católica do Rio Grande do Sul, Porto Alegre 90619-900, Brazil; daiana.schutz@edu.pucrs.br (D.M.S.); taina.rossi@edu.pucrs.br (T.R.); nathalia.albuquerque@edu.pucrs.br (N.S.d.A.); dalton.costa96@edu.pucrs.br (D.B.C.); jessica.machado.002@edu.pucrs.br (J.S.M.); natalia.sessegolo@edu.pucrs.br (N.S.); vitoria.bottega@edu.pucrs.br (V.R.B.); lewearick@gmail.com (L.E.W.-S.); 2 Faculty of Psychology, Universidad Católica de Valencia San Vicente Mártir, 46100 Burjassot, Valencia, Spain; 3 Department of Neuroscience, Mental Health and Sense Organs (NESMOS), Faculty of Medicine and Psychology, Sapienza University of Rome, 00185 Rome, Italy

**Keywords:** health promotion, mental health, aging, loneliness, COVID-19

## Abstract

The study focused on examining the relationship between well-being and various psychological factors such as loneliness, anxiety, depression, and stress, whilst also considering changes in lifestyle. A total of 108 elderly participants, with an average age of 70.38 years, were enrolled in this quantitative cross-sectional study. The research employed a battery of assessment tools including a Sociodemographic Data Questionnaire, Mini-Mental State Examination, Positive Mental Health Scale, Stress Perception Scale, Geriatric Anxiety Inventory, Geriatric Depression Scale (reduced version), Loneliness Scale, and International Physical Activity Questionnaire. Descriptive analysis was conducted in order to understand the distribution of scores across these variables, followed by the categorization of participants based on the reported alterations in eating and physical activity behaviors. Correlations between variables were assessed using Spearman correlation and an EBIC-LASSO network analysis. The findings indicated a potential detriment to the well-being of elderly individuals practicing social distancing, evidenced by heightened symptoms of loneliness, depression, anxiety, and stress, alongside the reported changes in dietary patterns and physical activity. The study underscores the importance of understanding the pandemic’s impact on the well-being of older adults and advocates for longitudinal investigations to delineate the evolving effects of social distancing measures across different phases of the pandemic.

## 1. Introduction

According to the World Health Organization (WHO), the COVID-19 virus induces an infectious ailment that typically results in mild or moderate respiratory complications, with a propensity for severe manifestations in individuals possessing pre-existing comorbidities [1]. Its primary mode of transmission is through respiratory droplets, predominantly derived from saliva or nasal secretions. Given its high transmissibility, the virus rapidly disseminated across multiple continents, precipitating a worldwide pandemic [2].

As the disease spread, several risk groups were identified, one of which was the elderly [1]. Social distancing measures were implemented in several countries to protect this and other more vulnerable groups. In Brazil, several forms of distancing were implemented in March 2020 [3]. These restrictive measures not only affected physical health, but also emotional and social health [4]. As the period of social distancing became longer, more severe symptoms of anxiety, depression, and stress were observed [5]. Society was exposed to several stressors, such as the increase in the number of infected cases, the extended period of social distancing [6], and the increase in SARS-CoV-2 variants such as Alpha, Beta, Delta, and Omicron [7].

Although the aging process can introduce alterations in both physical and mental well-being, it is imperative to acknowledge the considerable inter-individual variability. Aging per se does not unequivocally ensure the manifestation of deleterious effects, and a substantial number of older adults sustain commendable levels of physical and mental health [8]. According to these authors, the stress-response paradigm provides a valuable framework for defining key elements: a stressor, organism response, and resultant outcome. Stressors can range from acute events like viral infections, such as the COVID-19 outbreak, to chronic conditions, or psychological and physical challenges.

Regular physical activity is essential for keeping the body functioning properly and preventing several chronic non-communicable diseases, such as cardiovascular disease, type 2 diabetes, obesity, and some types of cancer. Physical activity also helps to strengthen the immune system, making the body more resistant to infections and diseases. The variety of movements and intensities provided by physical activity promotes joint mobility, flexibility, and balance, reducing the risk of falls and injuries, especially in the elderly. In addition to the physical benefits, regular physical activity also plays a key role in mental health, helping to reduce symptoms of anxiety, depression, and stress, improving mood, and promoting a general sense of well-being. It is therefore essential to encourage the adoption of an active lifestyle and integrate physical activity as an essential part of health promotion strategies across all age groups and communities [9].

In this scenario, lifestyle, such as engaging in physical exercise, is recommended for all healthy adults, particularly the elderly, as it serves as a preventive measure against chronic diseases and facilitates the maintenance of independent performance of daily activities [10]. However, as they belonged to a risk group, the elderly tended to be affected by social distancing in two main ways. Firstly, some elderly people decided to impose their own social distancing measures, as they were afraid of being contaminated and developing severe symptoms of the disease or even dying. Secondly, younger family members of these elderly people limited their visits to their older family members for fear of transmitting the virus [11,12,13]. In any case, it is reasonable to postulate that the elderly would be more vulnerable to the negative effects of social distancing protocols.

One should also bear in mind that the restrictions associated with the pandemic, such as social isolation measures, disruptions in routine healthcare services, and increased stress related to the virus, may accentuate challenges for older individuals [6,14]. In this way, the elderly population subjected to social isolation may exhibit heightened manifestations of anxiety and an exacerbated fear response to the ongoing pandemic, leading to significant emotional distress [15]. Moreover, the implementation of restrictive measures aimed at curtailing the transmission of the novel coronavirus contributes to an escalation in feelings of loneliness among the elderly.

Loneliness, a recognized precursor to anxiety and depression in this demographic, is evidenced by previous studies [16,17,18,19]. Furthermore, loneliness has been established as a correlate of diverse physical and mental health issues, including but not limited to obesity, cardiovascular diseases, anxiety disorders, cognitive decline, Alzheimer’s disease, and mortality [5]. Recognizing and understanding these components are crucial for designing effective interventions and support systems aimed at promoting psychological well-being in the elderly during the challenging circumstances brought about by the named outbreak.

Well-being is a state of balance and harmony that encompasses different dimensions of an individual’s life, including physical, emotional, mental, social, and spiritual aspects. It is not just the absence of illness or problems, but involves a general sense of satisfaction, happiness, and personal fulfillment. Well-being is influenced by various factors, such as lifestyle, interpersonal relationships, working conditions, physical environment, mental health, and spirituality. It is a subjective and individual experience which can vary according to each person’s perceptions and values. In addition, well-being is not static and can be influenced by events and changes throughout life. Thus, promoting well-being involves adopting healthy practices and habits, cultivating positive relationships, seeking meaning and purpose in life, and developing coping skills to deal with the challenges of everyday life [9,20].

This study seeks to explore the relationship between well-being and indicators of loneliness, anxiety, depression, and stress, alongside potential alterations in activity among elderly individuals adhering to social distancing measures prompted by the COVID-19 pandemic. Furthermore, the study endeavors to ascertain whether any of these factors serve as predictors for well-being. The independent variable under investigation is lifestyle, whereas the dependent variables encompass well-being, symptoms of loneliness, anxiety, depression, and stress.

## 2. Materials and Methods

### 2.1. Procedure

This is a cross-sectional, web-based study with elderly people aged from 60 to 90 years and living in the southern region of Brazil during the COVID-19 isolation period. Participants were recruited via social media advertising (Instagram, Facebook, and WhatsApp), for the sake of convenience, using a snowball strategy.

The data was collected in interviews conducted during video calls carried out between October 2020 and June 2021, with an average duration of 2 h. The responses were recorded by the researchers on the Redcap platform. To be included in this study, participants were required to be 60 years old or over, speak Portuguese, be a Brazilian citizen, had responded to the reading of the free and informed consent form (via an audio recording), and agree to participate in the study, as well as having access to the internet. Participants who scored less than 15 on the Mini-Mental State Examination or did not complete all of the research tools were excluded.

### 2.2. Participants

**Figure 1:**
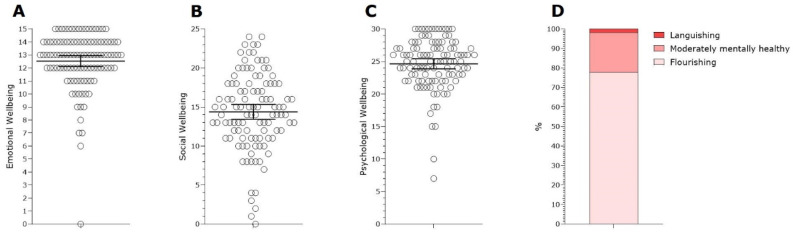
Scores distributions of emotional, social, and psychological well-being of all the participants. Scores distributions of MHC-SF factor scores of emotional, social, and psychological well-being of all the participants as well as a classification of flourishing and languishing mental health based on these scores. (**A**) Distribution of emotional wellbeing factor, which represents the positive effects of life satisfaction; (**B**) Distribution of social wellbeing factor, which represents connectivity and beliefs of affiliation to social groups; (**C**) Distribution of psychological well-being factor, which represents individual perception of personal growth and life purpose; (**D**) Classification of mental health indicator based on the subscales scores – presented in percentage. Data from (**A**–**C**) are presented in mean and standard deviation.

The sample consisted of elderly people living in the community. All the participants lived in urban areas. Although traditional calculations suggest needing around 138 participants based on variance, this study required a sample size of 28 to calculate the predictive network model due to the seven variables analyzed. With the sample size exceeding the number of unique parameters estimated by the model by 3.8 times, it was considered suitable for the analysis: 7 + (7 × 6/2) = 28. The sample size exceeds the number of unique parameters estimated by the predictive network model by 3.8 times, making it suitable for the analysis [21]. One participant was excluded for presenting a score below 15 in the MMSE. Therefore, the final sample size, consisted of 108 elderly people aged 60 to 90 years old, with a mean age of 70.3 ± 7.06. Sex distribution showed a greater percentage of women (n = 91, 87.5%) and the mean BMI was 27.93 ± 4.27. Most of the participants (n = 55, 52.9%) stated that they had at least a bachelor’s degree and 77 (74%) were retired. Figure 1 displays the distributions of the scores for emotional, social, and psychological well-being of all the participants.

### 2.3. Materials 

The online interview for this study was composed of a free and informed consent form, a sociodemographic section, and seven tools. Information regarding the questionnaire’s structure and scoring are detailed below:

The Free and Informed Consent Form: this document was read via an audio message sent to the participant. The participant’s response, accepting the offer of participation in this research, was also recorded in audio. A copy of the terms was sent to the participant via a WhatsApp message. These terms highlighted the objectives of the study, the non-financial reimbursement for participation, and the confidentiality of the data.

Sociodemographic data was used to characterize the sample and consisted of questions about age, sex, occupational status, education, and income. There were also questions related to changes in eating behavior and physical activity, in addition to adherence to social isolation. To assess the changes in eating behavior and physical activity, the following questions were asked: Compared to the beginning of the pandemic: (1) Have you noticed a change in the amount of physical activity you do during the day? Answer options: No, Yes—I am doing fewer activities, or Yes—I am doing more activities; (2) Have you noticed any changes in your daily diet? Answer options: No or Yes (How?); and (3) Have you noticed a change in your eating habits (desire to eat)? Answer options: No, Yes—I am hungrier, or Yes—I do not feel as hungry as I used to. The Mini-Mental State Examination (MMSE)—telephone version [22] used in this study was adapted by [23] as a cognitive screening tool for the exclusion of elderly individuals with scores suggestive of dementia. The MMSE consists of reduced and adapted questions for applying by telephone. It comprises 22 questions that assess temporal orientation, spatial orientation, registration of three words, attention and calculation, recall of three words, and language.

The Positive Mental Health Scale [24] consists of 14 items answered on a six-point Likert scale, ranging from “never” (1) to “every day” (6). The tool has three subscales: (a) emotional well-being (evaluating the positive effects of satisfaction with life); (b) psychological well-being (individual perception of personal growth, purpose in life, and other characteristics related to personal development); and (c) social well-being (identifying beliefs of affiliation, connectivity, and compatibility of values with one’s social group). Various analyses, including item analysis, network analysis, exploratory factor analysis, evidence of convergent validity, and precision, were conducted to investigate the psychometric qualities of the scale, and indicate whether it was suitable for applying to the Brazilian population. It achieved a satisfactory reliability index with a Cronbach’s alpha coefficient of 0.96. This scale has behavior characteristic of the dimensional continuum construct.

The Perceived Stress Scale (EPS-10) [25] is a five-point Likert scale (1—never, 2—almost never, 3—sometimes, 4—almost always, and 5—always) that assesses the frequency with which an individual perceives the current context as a stressful situation, based on 10 items. It was validated for Brazilian Portuguese on a sample of elderly individuals, which determined that it was valid in terms of clarity and construction. It achieved a satisfactory reliability index, with a Cronbach’s alpha coefficient of 0.82. The Exploratory Factor Analysis of the scale revealed that there were two factors: items related to positive aspects and items related to negative aspects of stress.

The Geriatric Anxiety Inventory (GAI) [26] consists of 20 dichotomous statements: either agree (one point) or disagree (zero points). A score greater than 10 points indicates the elderly person has symptoms of anxiety. In relation to psychometric parameters, the GAI had a Cronbach’s alpha coefficient of 0.91 for the elderly population [27].

The Geriatric Depression Scale (GDS-15) [28] is used to identify depressive symptoms in the elderly. The reduced version of 15 dichotomous questions (Yes or No) was employed. The total score is the sum of the responses marked on the 15 items, with a possible range from zero to 15. Scores above 5 points indicate the presence of depression symptoms [29]. It has a Cronbach’s alpha coefficient of 0.94 [30].

The Loneliness Scale (UCLA-BR) [31] consists of 20 statements assessing loneliness on a Likert scale from 0 (never) to 3 (often). The scale had a Cronbach’s alpha coefficient of 0.94. The tool’s maximum score is 60 points. Interpretation cutoffs are as follows: 0 to 22 points, indicative of minimal loneliness; 23 to 35 points, indicative of mild loneliness; 36 to 47 points, indicative of moderate loneliness; and 48 to 60 points, indicative of severe loneliness [32].

The International Physical Activity Questionnaire (IPAQ) [33] assesses the weekly frequency and daily hours of physical activity. It evaluates sedentary behavior and physical activities of light, moderate, and vigorous intensity. In this study, we used the shortened version comprising eight questions. The data, in categories, was analyzed to determine the percentage of agreement (Correct Classification Median) and the Kappa coefficient. A significance level of *p* < 0.05 was adopted.

### 2.4. Ethical Procedures 

This study was approved by the Local Ethics Committee of the Pontifical Catholic University of Rio Grande do Sul under CAAE #37857620.4.0000.5336 and followed the ethical standards defined by Resolution 466/12 of the National Health Council of Brazil.

### 2.5. Data Analysis

The data was analyzed using the statistical software JASP 0.16.3. Demographic data is presented in terms of frequency and percentage for categorical variables and mean and standard deviation for continuous variables. After the groups were stratified according to changes in eating behavior and physical activity during lockdown, the assumption of data normality was checked using the Kolmogorov-Smirnov test which showed a non-parametric distribution of data. Therefore, a Mann–Whitney U test was performed, and the effect size was estimated by rank–biserial correlation. To investigate the bivariate association between variables, correlations were calculated using the Spearman coefficient. We used an Extended Bayesian Information Criteria (EBIC) for the network analysis, in combination with a Least Absolute Shrinkage and Selection Operator (LASSO) model in which edges are represented by partial correlations (Friedman). Thicker edges represent stronger partial correlation, controlling for all other correlations. Our data met the main assumptions to conduct a network analysis which include in-dependent observations, the presence of linear relationships and no missing data. Thicker edges represent a stronger partial correlation, controlling for all other correlations. Blue edges represent positive associations and red edges represent negative associations. Finally, we used ‘closeness’ (largest number of connections among all the possible ones) as an index of node centrality as well as node’s expected influence.

## 3. Results

**Figure 2:**
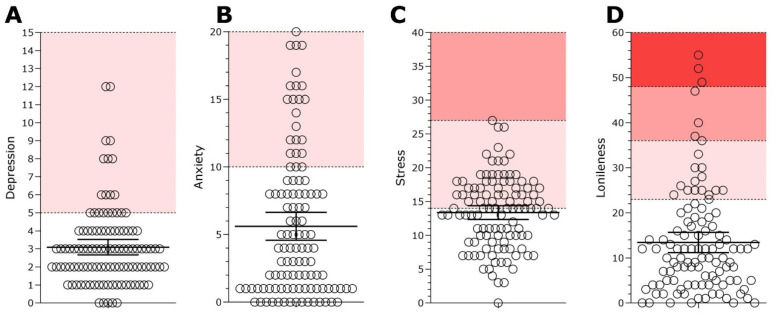
Score distributions of mental health indicators of the sample: depression (**A**), anxiety (**B**), stress (**C**), and loneliness (**D**). (**A**) (Depression): The white band represents participants without depressive symptoms (scores below 5), and the light red band indicates the presence of depressive symptoms (scores above 5). (**B**) (Anxiety): The white band indicates the absence of anxiety symptoms (scores below 10), and the light red band shows the presence of anxiety symptoms (scores above 10). (**C**) (Stress): The white band denotes the absence of significant stress symptoms (scores below 14). The light red band represents the presence of significant stress symptomatology (scores between 14 and 26), and the dark red band indicates severe stress symptoms (scores above 27). (**D**) (Loneliness): The bands represent increasing levels of loneliness, with white indicating minimal loneliness (scores from 0 to 22), followed by progressively darker shades of red for mild loneliness (23 to 35 points), moderate loneliness (36 to 47 points), and intense loneliness (48 to 60 points).

According to the MHC-SF criteria, 84 (77.8%) participants were classified as ‘flourishing’ regarding well-being, 22 (20.4%) participants were considered as moderately mentally healthy and only 2 (1.9%) were ‘languishing’ in terms of well-being (Figure 1D). In addition, Figure 2 displays the distributions of scores for the mental health indicators of the sample. A total of 20 participants (19.2%) scored above the threshold for depression (Figure 2A) and 22 participants (21.2%) scored above the threshold for anxiety (Figure 2B). In relation to perceived stress, the scores of 51 (47.22%) participants indicated low stress, whereas 56 (51.85%) participants were considered under moderate stress and only 1 (0.93%) participant was considered to be under high perceived stress (Figure 2C). Furthermore, in relation to reported loneliness, 87 (80.55%) participants presented scores indicating a low degree of loneliness, 14 (12.96%) participants presented scores indicating a moderate degree of loneliness, 4 (3.7%) participants scored a moderately high degree of loneliness and 3 (2.79%) participants scored a high degree of loneliness (Figure 2D). These results suggest that most of the sample (77.8%) had high levels of well-being during the pandemic, however it is not possible to generalize these results for the entire population. Around 20% of participants showed symptoms of depression, anxiety and loneliness during the pandemic. In terms of stress, more than 50% of participants reported moderate levels of perceived stress.

In addition to well-being and mental health indicators, participants were asked about any changes to eating behavior or physical activity during lockdown. This data is presented in Table 1, in which 40 participants (37.03%) reported eating more after lockdown and 71 (65.74%) reported doing less physical activity after lockdown.

**Figure 3:**
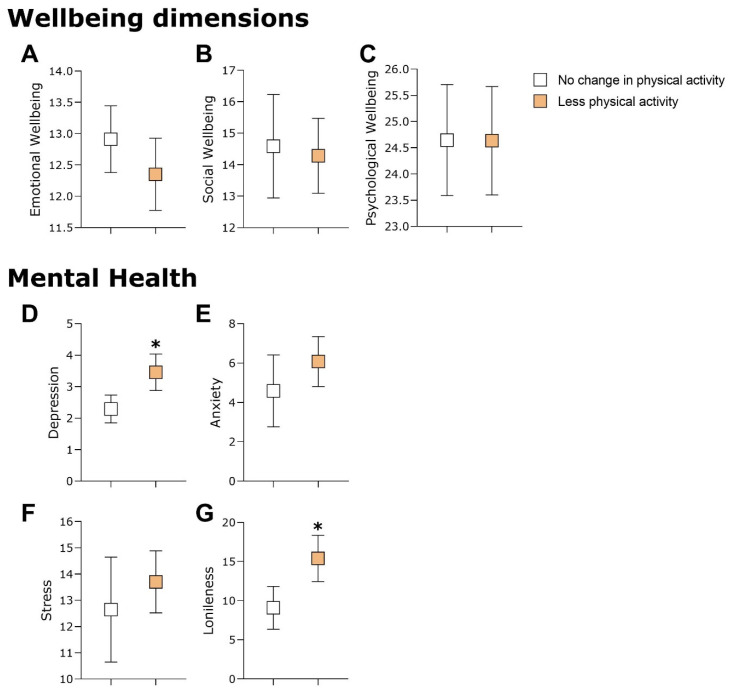
This figure illustrates the comparison between participants who did not change their physical activity levels and those who decreased them during the lockdown. (**A**–**C**) depict these groups in terms of wellbeing characteristics. (**D**–**G**) present comparisons of the same groups regarding mental health aspects such as depression, anxiety, stress, and loneliness. Significant differences were found only in depression (**D**) and loneliness (**G**), and are marked with an asterisk (*).

**Figure 4:**
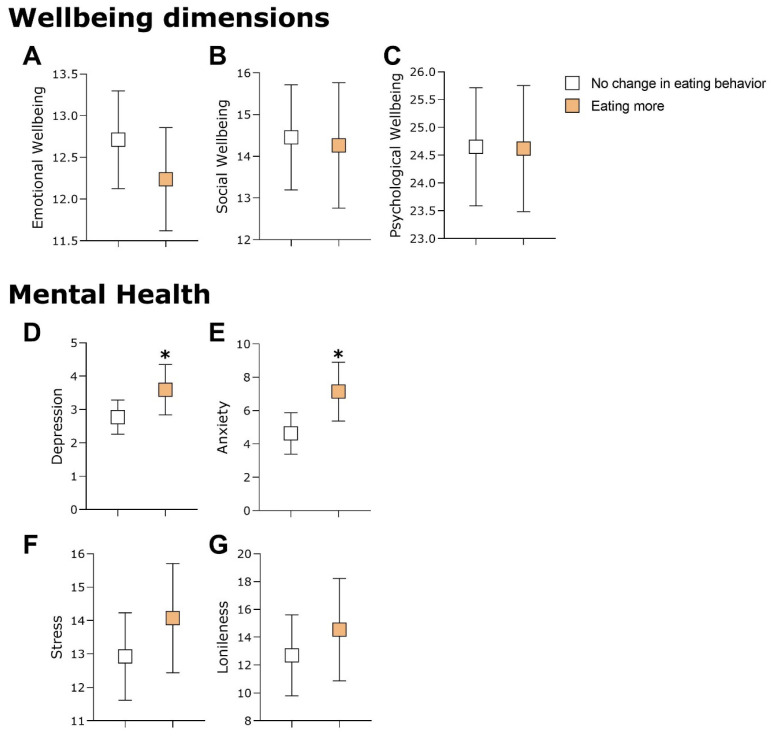
Comparison between participants who did not change their eating behaviors and those who increased them during the lockdown. (**A**–**C**) show groups comparisons in relation to emotional, social and psychological wellbeing, respectively. (**D**–**G**) present comparisons of mental health indicator of depression, anxiety, stress, and loneliness, respectively. Significant differences (*p* < 0.05) were found in both anxiety and depression indicated with an asterisk (*). Data are presented in mean and standard deviation.

Follow-up analyses were conducted after the groups had been stratified in terms of changes in eating and physical activity behaviors and comparing the well-being and mental health indicators of the groups. Figure 3 shows that the participants who did less physical activity during lockdown presented higher scores for depression and loneliness compared to the participants who did not report any changes in physical activity (in Figure 3D: U = 1.592, *p* = 0.025, and rrb = −0.266; and in Figure 3G: U = 1.648, *p* = 0.010, and rrb = −0.310). When the sample was stratified by changes to eating behavior, those participants who started eating more during lockdown presented higher depression and anxiety scores than those participants who reported no changes to eating behavior (in Figure 4D: U = 1.720, *p* = 0.032, and rrb = −0.241; and in Figure 4E: U = 1.772, *p* = 0.014, and rrb = −0.278). These results suggest that participants who did less physical activity during lockdown had more symptoms of depression and loneliness. In addition, the elderly people who started eating more after lockdown showed more symptoms of depression and anxiety.

**Figure 5:**
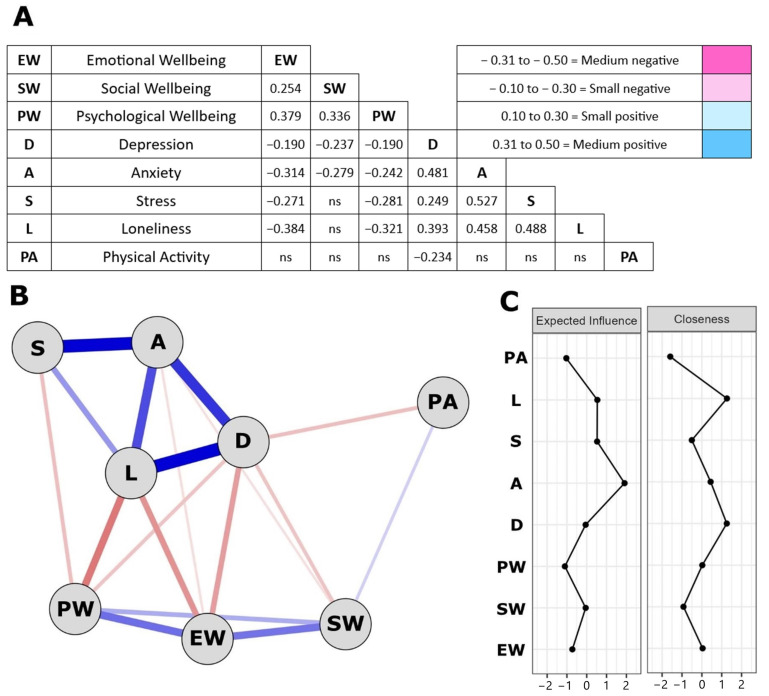
The association between well-being dimension, mental health indicators, and physical activity levels through a network analysis approach. Correlations (**A**), network analysis (**B**) and Centrality scores (**C**).

The associations between the well-being dimension, the mental health indicators, and the physical activity levels, as well as the results from the network analysis are presented in Figure 5. The well-being scores had small to medium positive correlation scores between them, and the mental health indicators had a significant positive correlation with the scores for depression, anxiety, and stress, with the exception of depression and stress which showed a nominal positive correlation. The well-being scores had small to medium negative associations with mental health indicators and loneliness, except for social well-being with stress and loneliness. There was a small negative correlation between physical activity and depression scores (Figure 5A).

The network analysis is presented in Figure 5B. Notably, the variables that presented higher than expected influence (EI) were anxiety (EI = 1.907), loneliness (EI = 0.534), and stress (EI = 0.524), which suggests that these variables, when activated, contribute a larger amount to the network. In addition, closeness was used to investigate which variables had the largest number of connections possible. Loneliness (1.263), depression (1.254), and anxiety (0.442) are the nodes with the highest closeness scores, which suggests that, when activated, they can influence a greater number of variables because they have a higher number of connections in the network. The centrality scores are presented in Figure 5C.

## 4. Discussion

The main objective of this study was to investigate the relationship between well-being and symptoms of loneliness, anxiety, depression, and stress as well as any changes in eating behavior or physical activity by elderly people who adopted social distancing due to the COVID-19 pandemic. In addition, this study also sought to investigate whether any of these variables are predictors of well-being. The main findings show a negative relationship between symptoms of loneliness, anxiety, depression, and stress and the well-being of elderly people who were social distancing during the COVID-19 pandemic. These symptoms have also been described in other studies on the impacts of the pandemic on the mental health of the general population [12,34,35,36,37].

Other studies conducted during COVID-19 indicate that social isolation increased the symptoms of loneliness in this population [38,39,40]. This increase may have been due to the need to avoid physical contact with others [38], as well as difficulties communicating with family and friends through technology (such as video calls or messaging applications) [39]. These factors could have increased the negative impact of social distancing on the feelings of loneliness in the elderly and diminish their sense of well-being, considering that loneliness is not just about the times when one is alone, but being alone increases the likelihood that the phenomenon of loneliness will take the form of suffering [41].

The findings of this research showed that symptoms of depression and anxiety are related to poorer well-being in the elderly during social distancing. This damage to the well-being of the elderly can make it difficult to make positive changes, resulting in a devaluation of the subject and it can make it difficult to see new ways to look at the future. Therefore, this can make the symptoms of depression worse [9]. Consequently, both forms of depression can lead to poor well-being, and poor well-being can lead to more depressive symptoms.

In addition, some of the participants presented elevated symptoms of anxiety and depression. There is no consensus in the literature on how these symptoms affect the elderly population. A study conducted with elderly people over 75 years of age showed that the participants exhibited low levels of both the symptoms of depression and of anxiety [38]. On the other hand, another study revealed that a large proportion of elderly people had depressive and anxiety symptoms during the pandemic [42]. Those elderly people who were single, widowed, divorced, or who lived alone were more likely to report that their symptoms of depression and anxiety became worse after social distancing due to COVID-19 [39]. According to the literature, the elderly population tends to be the age group that is most affected by symptoms of depression due to changes caused by aging, such as retirement, loss of loved ones, and medical issues [43,44]. One possible explanation for this discord could be the resilience of some elderly people whom developed ways to cope and adapt to adverse situations, such as social distancing. This is reflected in the findings of the study by [45], in which older participants adapted better to the social isolation of COVID-19.

In relation to stress, elderly people who were social distancing and suffered from this symptom also presented a lower standard of well-being. This stress may be related to the risk of infection or restrictions on social interaction and leisure activities during the pandemic [46,47]. In addition, the elderly may have been under stress, not only due to concerns for their own health, but also due to the risk of contamination with family and friends [47]. All of these anxieties can have a negative impact on the well-being of the elderly, particularly as the greater the stress, the more damage it causes to those who are under it. These changes can lead to the emergence of many diseases and an increase in the risk of immune system damage, whereas stress also tends to aggravate diseases, notably in the elderly [48].

Most participants reported that they undertook physical exercise regularly. This result was also observed in another study on the elderly during COVID-19 [38]. However, most participants also stated that they had been doing less physical activity since the beginning of social distancing. This is a worrying finding since habitual exercise is an important factor for regulating and preserving the mental and physical health of the elderly. If there is a decline in functional abilities, this can, consequently, present a weakening of functions, which leads to them becoming more susceptible to contracting diseases and developing health complications [10]. Physical exercise also encourages greater self-esteem and self-confidence in the elderly [37]. It also helps to maintain other physiological and psychological functions, such as those affected by multiple sclerosis and Alzheimer’s disease [49]. The reasons why the elderly in this study were undertaking less physical exercise may have been because they had been participating in these activities in places such as parks, squares, or gyms, and many of these places were closed due to the pandemic. Moreover, because they belong to a risk group and need to maintain strict social distancing, the alternative physical activity was to exercise at home, or attend lessons by video, which is not always possible for the elderly.

In terms of limitations of this study, it should be noted that the data was collected online and by snowball methods. Another limitation was the fact that the required sample size was not reached, which was 138 elderly people. The final sample consisted of only 108 elderly people. As the data collection period was during the pandemic, the elderly were mostly alone at home. This fact may have made it difficult to achieve the required sample size, as many elderly people do not know how to use WhatsApp video calls, requiring the help of another person. In addition to the limitations inherent to online interviewing, such as internet connection problems, Redcap system outages, and difficulties that some elderly people experience using cellular devices, it is important to point out that this collection method makes it impossible to gain access to all the population. We can see this limitation in the sample, because most of the participants had completed higher education. This makes it difficult to generalize these results in relation to the general population. Another limiting factor in this research was the need for the elderly to have access to the instant messaging application necessary to make the video call, which may have influenced the recruitment of the participants. Furthermore, in relation to the answers, we must consider the social desirability of the elderly in their answers, which may affect the answers in a more positive way. However, there are also positive aspects in relation to applying an online survey to the elderly, such as the requirement to fully complete the data in order to continue, which prevents data gaps and typing errors from appearing in transcribed answers (which were transferred directly to the database).

In summary, the results of this research provide insights that support the stress–response paradigm in understanding the impact of social distancing measures on the well-being of elderly individuals during the COVID-19 pandemic [8]. The findings reveal that symptoms of depression and anxiety are significantly related to a decreased well-being among the elderly population practicing social distancing. Such compromised well-being can hinder their ability to enact positive changes, potentially leading to a sense of devaluation and a limited perception of future possibilities, thereby exacerbating depressive symptoms [6,14]. Notably, the prevalence of depressive and anxiety symptoms among the elderly during the pandemic varies in the literature, possibly due to factors such as resilience and adaptive coping mechanisms. Whereas some elderly individuals may adapt well to social isolation, others experience heightened stress, which correlates with lower levels of well-being. This stress may stem from concerns about infection risk, limitations in social interactions, or disruptions to leisure activities. Chronic stress can disrupt the body’s stress response system, leading to detrimental physiological changes such as reduced immunity and increased inflammation, thereby elevating the risk of disease and exacerbating existing health conditions, particularly in older adults. Additionally, the study highlights a decline in physical activity levels among elderly individuals during social distancing, which is concerning as regular exercise plays a vital role in maintaining both physical and mental health in this population. The closure of public exercise spaces and the need for strict social distancing measures likely contributed to this decline, underscoring the importance of accessible alternatives for physical activity tailored to the needs of elderly individuals during times of social upheaval, like during the COVID-19 pandemic.

## 5. Conclusions

The results of this research highlight a negative relationship between symptoms of loneliness, anxiety, depression, and stress and the well-being of the elderly population who were social distancing during the COVID-19 pandemic. The majority of participants presented indications of some degree of loneliness and some participants presented elevated symptoms of anxiety and depression. The elderly people who were social distancing presented more symptoms of stress and a lower standard of well-being. The majority of participants were doing less physical activity than they were doing at before social distancing measures were introduced. Being able to understand the effect of COVID-19 and the consequences of social distancing on the well-being of the elderly population is important for the development of intervention strategies for this population and to prevent symptoms from developing. It is suggested that future studies use a longitudinal design to verify the effects of the pandemic on the elderly at different stages of social distancing.

## Figures and Tables

**Table 1:** Changes in eating behavior and physical activity during lockdown.

Changes during Lockdown	Yes		No	
	n	%	n	%
Changes in eating behavior				
Eating more after lockdown	40	37.03	68	62.97
Changes in physical activity				
Less physical activity	71	65.74	37	34.26

## Data Availability

Data are contained within the article.

## References

[1] World Health Organization (2020). Similarities and Diferences between COVID-19 and Influenza. https://www.paho.org/pt/news/25-3-2020-similarities-and-differences-covid-19-and-influenza.

[2] Liu W., Yue X.-G., Tchounwou P.B. (2020). Response to the COVID-19 Epidemic: The Chinese Experience and Implications for Other Countries. Int. J. Environ. Res. Public Health.

[3] da Silva L.L.S., Lima A.F.R., Polli D.A., Razia P.F.S., Pavão L.F.A., Cavalcanti M.A.F.d.H., Toscano C.M. (2020). Medidas de distanciamento social para o enfrentamento da COVID-19 no Brasil: Caracterização e análise epidemiológica por estado. Cad. Saude Publica.

[4] Hammerschmidt K.S.d.A., Bonatelli L.C.S., de Carvalho A.A. (2020). Caminho da esperança nas relações envolvendo os idosos: Olhar da complexidade sobre pandemia da COVID-19. Texto Context.-Enferm..

[5] Schütz D.M., Borges L., Ferreira H.G., Irigaray T.Q. (2022). Relationship between loneliness and mental health indicators in the elderly during the COVID-19 pandemic. Psico-USF.

[6] Van Bavel J.J., Baicker K., Boggio P.S., Capraro V., Cichocka A., Cikara M., Crockett M.J., Crum A.J., Douglas K.M., Druckman J.N. (2020). Using social and behavioural science to support COVID-19 pandemic response. Nat. Hum. Behav..

[7] Maslo C., Friedland R., Toubkin M., Laubscher A., Akaloo T., Kama B. (2021). Characteristics and Outcomes of Hospitalized Patients in South Africa During the COVID-19 Omicron Wave Compared with Previous Waves. JAMA.

[8] Pascual-Leone A., Bartres-Faz D. (2021). Human brain resilience: A call to action. Ann. Neurol..

[9] de Oliveira D.S., Lima M.P., Ratto C.G., Rossi T., Baptista R.R., Irigaray T.Q. (2020). Avaliação de Bem-Estar Psicológico e Sintomas Depressivos em Idosos Saudáveis. Estud. Pesqui. Psicol..

[10] Camboim F.E.F., Nóbrega M.O., Davim R.M.B., Camboim J.C.A., Nunes R.M.V., Oliveira S.X. (2017). Benefícios da atividade física na terceira idade para a qualidade de vida. Rev. Enferm. UFPE Line.

[11] Grolli R.E., Mingoti M.E.D., Bertollo A.G., Luzardo A.R., Quevedo J., Réus G.Z., Ignácio Z.M. (2021). Impact of COVID-19 in the Mental Health in Elderly: Psychological and Biological Updates. Mol. Neurobiol..

[12] de Oliveira V.V., de Oliveira L.V., Rocha M.R., Leite I.A., Lisboa R.S., de Andrade K.C.L. (2021). Impactos do isolamento social na saúde mental de idosos durante a pandemia pela COVID-19. Braz. J. Health Rev..

[13] Qiu J., Shen B., Zhao M., Wang Z., Xie B., Xu Y. (2020). A nationwide survey of psychological distress among Chinese people in the COVID-19 epidemic: Implications and policy recommendations. Gen. Psychiatry.

[14] Kluge H.H.P. (2020). Statement–Older People Are at Highest Risk from COVID-19, but All Must Act to Prevent Community Spread. World Health Organization Europe. https://www.who.int/europe/news/item/03-04-2020-statement-older-people-are-at-highest-risk-from-covid-19-but-all-must-act-to-prevent-community-spread.

[15] Júnior F.E.D.N., Tatmatsu D.I.B., De Freitas R.G.T. (2020). Ansiedade em idosos em tempos de isolamento social no brasil (COVID-19). Rev. Bras. Análise Comport..

[16] Barroso S.M., Baptista M.N., Zanon C. (2018). Solidão como variável preditora na depressão em adultos. Estud. Interdiscip. Psicol..

[17] Moret-Tatay C., Murphy M. (2022). Anxiety, resilience and local conditions: A cross-cultural investigation in the time of COVID-19. Int. J. Psychol..

[18] Santini Z.I., Jose P.E., Cornwell E.Y., Koyanagi A., Nielsen L., Hinrichsen C., Meilstrup C., Madsen K.R., Koushede V. (2020). Social disconnectedness, perceived isolation, and symptoms of depression and anxiety among older Americans (NSHAP): A longitudinal mediation analysis. Lancet Public Health.

[19] Simard J., Volicer L. (2020). Loneliness and Isolation in Long-term Care and the COVID-19 Pandemic. J. Am. Med. Dir. Assoc..

[20] Zanatta C., De Santana C.M.L., Domingos L.F., Campos L.A.M., Santos M.C.F. (2021). Bem-estar psicológico e percepção de suporte social: Uma análise sobre idosos e a pandemia COVID 19. Rev. Valore.

[21] da Cunha Lemea D.E., da Costa Alvesa E.V., Lemosb VD C.O., Fattoria A. (2020). Análise de redes: Uma abordagem de estatística multivariada para pesquisas em ciências da saúde. Geriatr. Gerontol. Aging.

[22] Roccaforte W.H., Burke W.J., Bayer B.L., Wengel S.P. (1992). Validation of a Telephone Version of the Mini-Mental State Examination. J. Am. Geriatr. Soc..

[23] Camozzato A.L., Kochhann R., Godinho C., Costa A., Chaves M.L. (2011). Validation of a telephone screening test for Alzheimer’s disease. Aging Neuropsychol. Cogn..

[24] Machado W.d.L., Bandeira D.R. (2015). Escala de Saúde Mental Positiva: Validação da Mental Health Continuum-Short Form. Psico-USF.

[25] Luft C.D.B., Sanches S.d.O., Mazo G.Z., Andrade A. (2007). Versão brasileira da Escala de Estresse Percebido: Tradução e validação para idosos. Rev. Saude Publica.

[26] Massena P.N., De Araújo N.B., Pachana N., Laks J., De Pádua A.C. (2015). Validation of the Brazilian Portuguese version of geriatric anxiety inventory–GAI-BR. Int. Psychogeriatr..

[27] Pachana N.A., Byrne G.J., Siddle H., Koloski N., Harley E., Arnold E. (2007). Development and validation of the Geriatric Anxiety Inventory. Int. Psychogeriatr..

[28] Almeida O.P., Almeida S.A. (1999). Short versions of the geriatric depression scale: A study of their validity for the diagnosis of a major depressive episode according to ICD-10 and DSM-IV. Int. J. Geriatr. Psychiatry.

[29] Paradela E.M.P., Lourenço R.A., Veras R.P. (2005). Validação da escala de depressão geriátrica em um ambulatório geral. Rev. Saude Publica.

[30] Fountoulakis K.N., Tsolaki M., Iacovides A., Yesavage J., O’hara R., Kazis A., Ierodiakonou C. (1999). The validation of the short form of the Geriatric Depression Scale (GDS) in Greece. Aging Clin. Exp. Res..

[31] Barroso S.M., de Andrade V.S., Midgett A.H., de Carvalho R.G.N. (2016). Evidências de validade da Escala Brasileira de Solidão UCLA. J. Bras. Psiquiatr..

[32] Barroso S.M., Andrade V.S.D., Oliveira N.R.D. (2016). Escala Brasileira de Solidão: Análises de Resposta ao Item e definição dos pontos de corte. J. Bras. Psiquiatr..

[33] Matsudo S., Araújo T., Matsudo V., Andrade D., Andrade E., Oliveira L.C., Braggion G. (2001). Questionário internacional de atividade física (ipaq): Estudo de validade e reprodutibilidade no Brasil. Atividade Física Saúde.

[34] Armitage R., Nellums L.B. (2020). COVID-19 and the consequences of isolating the elderly. Lancet Public Health.

[35] Flint A.J., Bingham K.S., Iaboni A. (2020). Effect of COVID-19 on the Mental Health Care of Older People in Canada. Int. Psychogeriatr..

[36] Meng H., Xu Y., Dai J., Zhang Y., Liu B., Yang H. (2020). Analyze the psychological impact of COVID-19 among the elderly population in China and make corresponding suggestions. Psychiatry Res..

[37] Mehra A., Rani S., Sahoo S., Parveen S., Singh A.P., Chakrabarti S., Grover S. (2020). A crisis for elderly with mental disorders: Relapse of symptoms due to heightened anxiety due to COVID-19. Asian J. Psychiatry.

[38] Brown L., Mossabir R., Harrison N., Brundle C., Smith J., Clegg A. (2020). Life in lockdown: A telephone survey to investigate the impact of COVID-19 lockdown measures on the lives of older people (≥75 years). Age Ageing.

[39] Robb C.E., de Jager C.A., Ahmadi-Abhari S., Giannakopoulou P., Udeh-Momoh C., McKeand J., Price G., Car J., Majeed A., Ward H. (2020). Associations of Social Isolation with Anxiety and Depression During the Early COVID-19 Pandemic: A Survey of Older Adults in London, UK. Front. Psychiatry.

[40] Van Tilburg T.G., Steinmetz S., Stolte E., Van Der Roest H., De Vries D.H. (2020). Loneliness and mental health during the COVID-19 pandemic: A study among Dutch older adults. J. Gerontol. Ser. B.

[41] Ribeiro S.C., Ramos J.B.S. (2020). Elderly person loneliness in pandemic times. Res. Soc. Dev..

[42] Parlapani E., Holeva V., Nikopoulou V.A., Sereslis K., Athanasiadou M., Godosidis A., Stephanou T., Diakogiannis I. (2020). Intolerance of Uncertainty and Loneliness in Older Adults During the COVID-19 Pandemic. Front. Psychiatry.

[43] Guimarães L.D.A., Brito T.A., Pithon K.R., Jesus C.S.D., Souto C.S., Souza S.J.N., Santos T.S.D. (2019). Depressive symptoms and associated factors in elderly long-term care residents. Cienc. Saude Coletiva.

[44] Medeiros G.L.d.F., Toledo M., de Sousa M.N.A. (2022). Intervenções medicamentosas e depressão em idosos: Estudo em unidade básica de saúde da paraíba. Temas Saúde.

[45] Morales-Vives F., Dueñas J.M., Vigil-Colet A., Camarero-Figuerola M. (2020). Psychological Variables Related to Adaptation to the COVID-19 Lockdown in Spain. Front. Psychol..

[46] Heid A.R., Cartwright F., Wilson-Genderson M., Pruchno R. (2020). Challenges Experienced by Older People During the Initial Months of the COVID-19 Pandemic. Gerontol..

[47] Nimrod G. (2020). Changes in Internet Use when Coping with Stress: Older Adults during the COVID-19 Pandemic. Am. J. Geriatr. Psychiatry.

[48] Antunes J. (2019). Estresse e doença: O que diz a evidência?. Psicol. Saúde Doenças.

[49] Braga V.E.G., de Almeida K.C., Amâncio N.d.F.G. (2021). Exercícios físicos em idosos com doença de alzheimer: Uma revisão dos benefícios cognitivos e motores. Braz. J. Health Rev..

